# Spatiotemporal changes and degradation early-warning of key ecosystem services in China from 2015 to 2020

**DOI:** 10.1038/s41598-026-46005-y

**Published:** 2026-03-27

**Authors:** Shuo Dong, Hongen Hu, Guangliang Jia, Tianyi Cai

**Affiliations:** College of Landscape Architecture, Henan Agricultural University, No. 63 Nongye Road, Jinshui District, Zhengzhou City, 450002 Henan Province China

**Keywords:** Key ecosystem service, Degradation early warning, GIS, County-level scale, Ecological civilization, Ecology, Ecology, Environmental sciences, Environmental social sciences

## Abstract

Change assessment and degradation early-warning of Key Ecosystem Services (KES) provide a vital scientific foundation for addressing ecological degradation challenges and optimizing ecological governance policies. Counties are the fundamental unit for spatial governance and ecological policy implementation in China. However, the spatiotemporal dynamics and degradation risks of KES at this scale have not been systematically assessed in the context of ecological civilization construction. Focusing on the critical stage of China’s ecological civilization construction from 2015 to 2020, this study took all counties nationwide as the basic units to systematically analyze the spatiotemporal dynamics of three KES: water conservation (WC), soil conservation (SC), and windbreak and sand fixation (WSF). Furthermore, a fact-based ecological degradation early-warning model was developed to enable the precise identification of degraded areas and their respective warning levels. The results showed that: (1) From 2015 to 2020, the three KES nationwide exhibited a trend of overall stability coupled with a slight increase. Spatially, high-value zones for WC were primarily concentrated in the Qinghai-Tibet Plateau (QTP), southern mountainous regions, and key forest zones of Northeast China. High-value zones for SC were predominantly distributed across the Loess Plateau, QTP, Yunnan-Guizhou Plateau, and southeastern hilly and mountainous regions. WSF, in contrast, were highly concentrated in the arid and semi-arid regions of Northern China and the QTP. (2) The ecological degradation early-warning results indicate that 48.96% of counties nationwide were in a “no alert” state, while the remaining counties exhibited varying degrees of functional degradation. Among these, counties under light, moderate, and severe alerts accounted for 36.97%, 12.26%, and 1.83%, respectively. Severe-alert areas were mainly distributed in the extremely arid regions of Northwest China and the karst mountains of Southwest China, largely driven by the substantial degradation of SC functions. Based on these alert types and priority regions, this study proposes tiered ecological governance policy recommendations. Our proposed early-warning framework facilitates the intuitive and efficient identification of county-level KES degradation risks. Thus, the findings offer a scientific foundation for formulating targeted strategies for ecological conservation and restoration in territorial spatial planning.

## Introduction

Ecosystem Services (ES) serve as vital conduits between natural ecosystems and human well-being. These services provide the essential material foundation for human survival and represent integral components of Earth’s life-support systems^1–3^. However, the UN Millennium Ecosystem Assessment reports that anthropogenic activities have significantly degraded approximately 75% of terrestrial ecosystems and 66% of marine environments globally^4^. This systemic degradation directly jeopardizes human well-being and sustainable development through the persistent diminishment of ES^5,6^. Consequently, establishing robust dynamic monitoring and early-warning frameworks for ES is imperative to mitigate ecosystem degradation and facilitate the attainment of the UN 2030 Sustainable Development Goals (SDGs).

As a pivotal participant in global ecological governance, China warrants particular attention regarding the spatiotemporal evolutionary trajectory of its KES. Since the initiation of the Reform and Opening-up, the nation’s accelerated industrialization and urbanization have propelled economic expansion while simultaneously inducing substantial ecological costs, notably the impairment of critical service functions^7,8^. In response to these challenges, the Chinese government has implemented a series of magnificent ecological restoration projects since the 1980s—most notably the Grain-for-Green Program and the Natural Forest Conservation Program—which have yielded substantial ecological dividends^9,10^. Furthermore, since the 18th National Congress of the CPC, “Ecological Civilization” has been institutionalized as a fundamental national strategy. Consequently, the synergy between ecological conservation and high-quality development has emerged as a central pillar of the national governance framework^11^. Notwithstanding the notable success of these restoration initiatives, the spatiotemporal heterogeneity of China’s ES remains profoundly complex under the synergistic pressures of climate change and anthropogenic activities^12,13^. Consequently, a systematic evaluation of ES dynamics during the pivotal period of ecological civilization construction (2015–2020) is essential. Precisely identifying risk hotspots and early-warning thresholds for ecological degradation not only elucidates the efficacy of national conservation strategies but also provides critical evidence-based support for optimizing territorial spatial governance and ecological management policies.

Existing literature has extensively assessed a range of ecosystem services, such as WC, WSF, SC, carbon sequestration, and biodiversity maintenance^14–17^. WC, SC, and WSF are directly linked to core ecological issues in China, such as water security, soil conservation, and desertification control^18–20^. They also serve as key indicators for China’s main functional zoning and ecological protection red line demarcation^21,22^. KES assessments have largely followed two main methodological pathways: monetary valuation and biophysical quantification. The monetary valuation method aggregates diverse services through monetization^2,23^, yet its inherent subjectivity in coefficient attribution limits its capacity to capture the spatiotemporal granularity of ES dynamics across large scales^24^. In contrast, the material quality assessment method excels in revealing ecosystem service diversity through its objectivity and process simulation advantages^25,26^. Specifically, WC is frequently characterized by the water storage method, which evaluates the regulation capacity of ecosystems^27^. SC is typically quantified using the Revised Universal Soil Loss Equation (RUSLE) by calculating the discrepancy between potential and actual soil erosion^15,28^. Similarly, WSF assessments employ the Revised Wind Erosion Equation (RWEQ) to measure the mitigation of wind erosion by vegetation cover^17,29^. Due to its spatially explicit outputs, robust parameterization, and operational efficiency, the INVEST model has been widely adopted for multi-service assessments^30,31^. Leveraging these methodological frameworks, an extensive body of literature has elucidated the spatiotemporal heterogeneity of China’s ecosystem services across multiple spatial hierarchies. These include watershed scales^14,32^, urban agglomerations^33,34^, and administrative levels ranging from provinces^35,36^ and counties^37^ down to high-resolution grids^38,39^. Regarding specific KES categories, the WC function exhibits a distinct east-high, west-low spatial gradient^18^, while the SC function follows a south-high, north-low distribution^40,41^. In contrast, the WSF function is predominantly concentrated across western and northern China^17,42^. In terms of temporal dynamics, China’s ecosystem services have shown an overall improving trend^18,40,43^. Significant recovery is evident across most regions—including the seven major river basins, key ecological function zones, and national parks^12,44,45^. However, localized degradation persists in specific areas, highlighting the spatiotemporal complexity of ES variations^18,46^.

Research on early-warning systems for ecosystem services has primarily advanced along two paradigms. The first involves integrating ES with ecological risk and ecosystem health indicators to develop multidimensional risk assessment frameworks, which are subsequently validated through empirical investigations^47,48^. For instance, some scholars have integrated diverse ES categories into socio-ecological vulnerability and risk assessments for delta regions^49^. Others have diagnosed the holistic condition of ecosystem services by elucidating the causal linkages between ecosystem health, ecological risks, and ES provision within watershed systems^50^. The second paradigm focuses on future scenario simulations to anticipate potential degradation risks^51^. For instance, researchers have coupled land-use projections with the Invest model to evaluate ES fluctuations across diverse scenarios^52^. Furthermore, proactive ecological security early-warning frameworks have been developed by integrating historical trajectories with future-oriented projections^53^.

While the aforementioned studies have established a robust foundation for the monitoring and early-warning of China’s KES, certain methodological and empirical limitations persist, particularly when scrutinized within the pivotal period of Ecological Civilization development (2015–2020). Regarding the change assessment of KES, current literature remains largely constrained by either static cross-sectional analyses or retrospective investigations of earlier periods. Consequently, there is a conspicuous lack of high-frequency dynamic tracking during the pivotal policy window of 2015–2020, which hampers an accurate appraisal of the actual efficacy of Ecological Civilization initiatives. Furthermore, although counties constitute the basic administrative units for spatial governance and ecological policy execution in China^16^, systematic nationwide assessments of these units have not received sufficient attention. Regarding KES degradation early-warning, current risk assessment frameworks frequently resort to indicator superimposition, which may marginalize the direct signals of intrinsic KES fluctuations. Although scenario-based forecasting provides a forward-looking perspective, the inherent uncertainties associated with complex simulations constrain their efficacy for localized, county-level management. Consequently, developing a robust and operationally efficient early-warning system—calibrated for the county scale and capable of capturing empirical change signals—has emerged as an imperative research priority for supporting evidence-based ecological governance.

Focusing on the crucial period of China’s ecological civilization construction (2015–2020), this study takes all counties nationwide as the basic units to systematically analyze the spatiotemporal dynamics of three KES: WC, SC, and WSF. Furthermore, a fact-based ecological degradation early-warning model is developed to enable the precise identification of degraded areas and their respective warning levels. The specific objectives are to: (1) formulate a research framework for the change assessment and early-warning of KES; (2) reveal the spatiotemporal patterns and variation characteristics of KES; (3) identify the degraded areas of KES and their respective warning levels; (4) propose policy recommendations for tiered ecological governance tailored to different alert types and key regions. This research aims to provide evidence-based insights into the efficacy of China’s ecological governance and offer a scientific foundation for optimizing territorial spatial restoration and conservation strategies.

## Materials and methods

### Study area

**Fig. 1:**
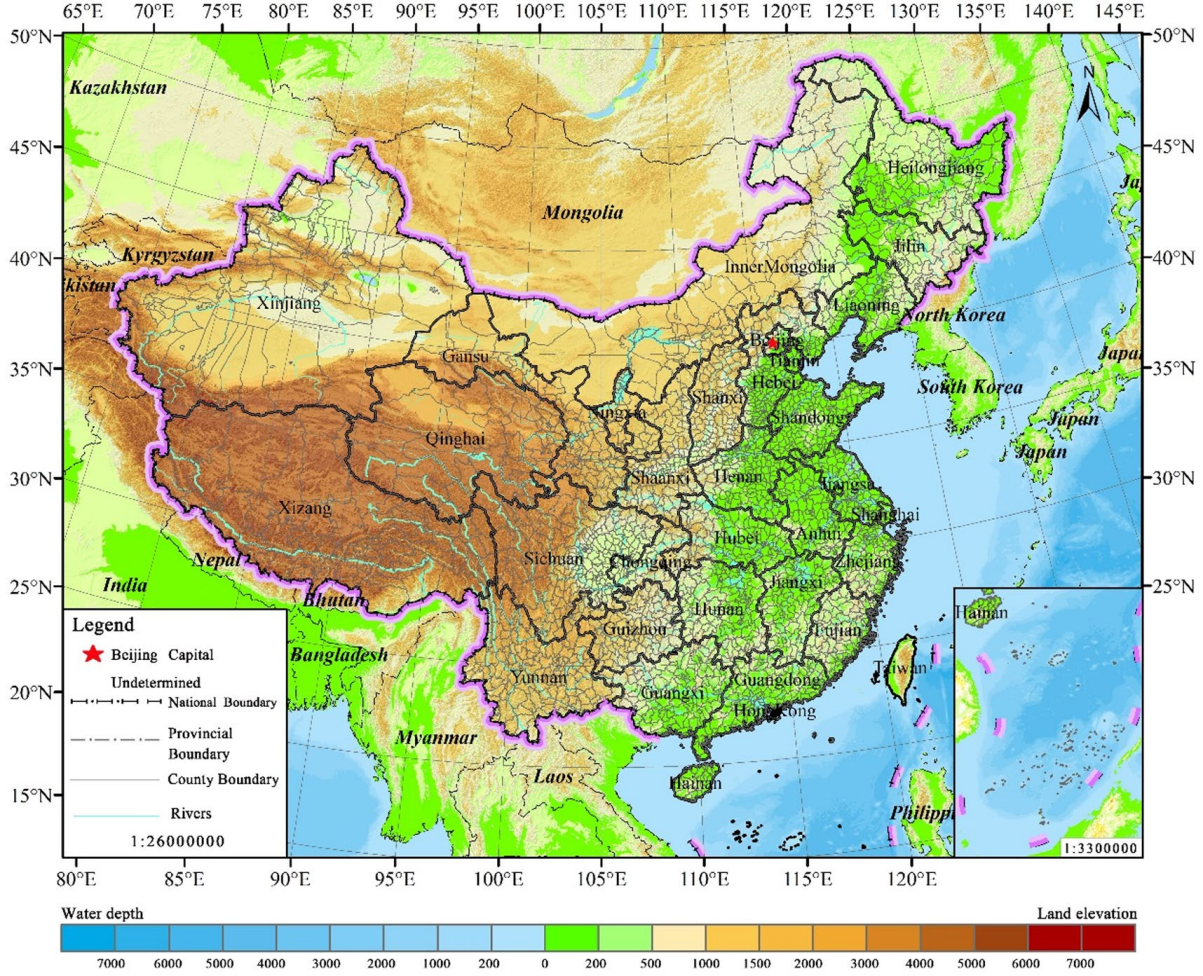
Overview of the study area. (The map was generated using ArcGIS 10.8).

Characterized by a vast territory and complex geomorphology, China exhibits a stepped topographic configuration that ascends progressively from the eastern coastal plains to the Qinghai-Tibet Plateau (QTP) in the west. The nation’s climatic spectrum is equally diverse, spanning tropical to cold-temperate zones and comprising monsoon, continental, and unique plateau-mountain climates. This intricate coupling of topographic relief and climatic variability fundamentally shapes the spatial heterogeneity of ecosystems, providing the biophysical template for the formation and distribution of KES. The WC functions are predominantly concentrated in the precipitation-rich southeastern mountains and critical river headwaters. The SC functions are centered in the southern hilly regions and the Loess Plateau, while WSF functions are primarily distributed across the ecological barrier zones of northern arid and semi-arid regions. Collectively, these three services constitute a vital defense line for national ecological security^21^. This study encompasses 31 provincial-level administrative units across mainland China (excluding Hong Kong, Macao, and Taiwan), utilizing 2847 county-level administrative districts as the fundamental analytical units (Fig. 1).

### Data sources

The 2015 and 2020 data products concerning China’s WC, SC, and WSF functions were provided on a fee-paying basis by the Resource and Environment Science Data Centre of the Chinese Academy of Sciences (https://www.resdc.cn) at a spatial resolution of 1 km. DEM data originates from the Geospatial Data Cloud (https://www.gscloud.cn/). Administrative vector boundaries were sourced from the 1:1,000,000 National Fundamental Geographic Database, provided by the National Catalogue Service for Geographic Information (https://www.webmap.cn/).

### Research framework

**Fig. 2:**
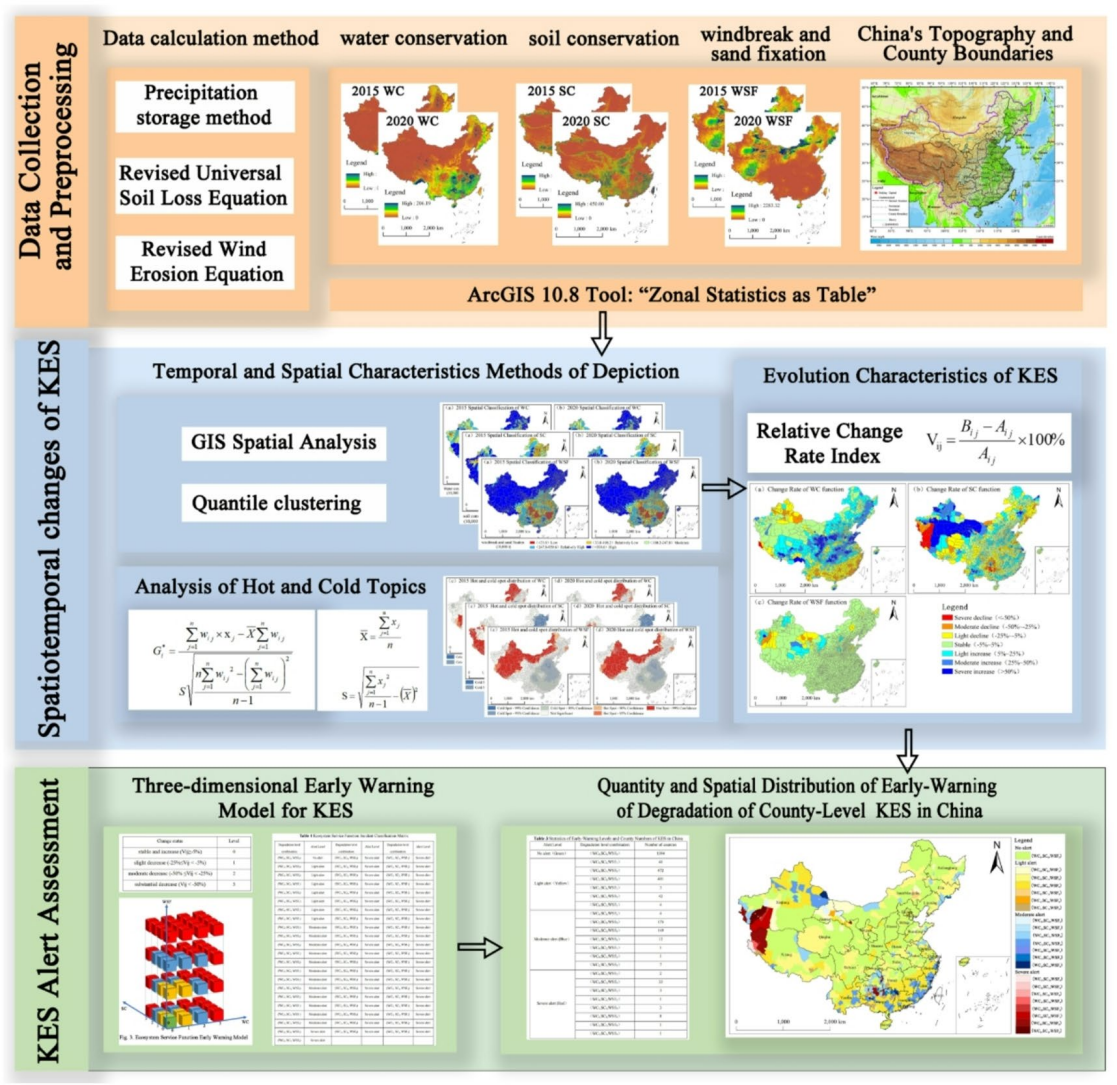
Research framework.

This study systematically examines the spatiotemporal variations and degradation early-warning of KES in China from 2015 to 2020. The research workflow is structured into the following three primary phases (Fig. 2): (1) Data collection and processing: Nationwide 1 km resolution data products for WC, WSF, and SC were acquired from the Chinese Academy of Sciences Resource and Environment Science Data Centre for 2015 and 2020 at a fee. These datasets were reprojected to ensure consistency with the spatial reference of China’s county-level administrative divisions. Subsequently, the Zonal Statistics tool within ArcGIS 10.8 was employed to calculate the aggregated functional totals for each county unit. (2) Spatiotemporal change analysis: Quantile clustering and Hotspot Analysis were employed to characterize the spatial patterns of KES at the county scale. Furthermore, relative change rate indices were utilized to quantitatively reveal the variation characteristics of KES during the monitoring period. (3) Degradation alert identification: A KES degradation early-warning model was developed based on relative change rates, complemented by an alert discrimination matrix. This framework categorizes KES status into four alert levels—no alert, light alert, moderate alert, and severe alert—at the county scale, followed by an analysis of their spatial distribution characteristics.

## Research methods

### Assessment methods for KES

#### Assessment method for WC

The WC function was quantified using the precipitation storage method^27^, which evaluates an ecosystem’s capacity to retain water by measuring its hydrological regulation effects. This approach conceptualizes the ecosystem’s water-holding capability relative to precipitation inputs and runoff mitigation.

#### Assessment method for SC

The SC was quantified using the Revised Universal Soil Loss Equation (RUSLE) by calculating the discrepancy between potential soil erosion (under extremely degraded conditions) and actual soil erosion (under current land-use and vegetation conditions)^15,28^. This approach defines SC capacity as the volume of soil loss prevented by the ecosystem.

#### Assessment method for WSF

The WSF functionality was calculated using the Revised Wind Erosion Equation (RWEQ) model. This approach quantitatively assesses potential wind erosion against actual erosion rates by comprehensively accounting for climatic conditions, vegetation cover, surface soil roughness, soil erodibility, and soil crusting. The difference between these two values represents the WSF service functionality^17,29^.

### Statistics on the total amount of KES at the county level

Based on national data concerning WC, SC, and WSF, alongside vector data of China’s county-level administrative divisions, the ‘Zonal Statistics as Table’ tool within ArcGIS 10.8 software was employed to extract and calculate the total values of the three KES categories for each county in 2015 and 2020 respectively.

### Spatial classification and Hotspot Analysis

Spatial hierarchical visualization is a commonly employed method for characterizing spatiotemporal patterns. To ensure the inter-annual comparability of the spatial grading results for WC, SC, and WSF, this study first pooled the functional totals from 2015 to 2020 for each category. Subsequently, the quantile classification method was applied to these pooled datasets to categorize each function into five hierarchical levels: low, relatively low, moderate, relatively high, and high. Finally, the spatial distribution of these classification results was mapped using ArcGIS 10.8. Furthermore, this study employs Hotspot Analysis (Getis-Ord Gi) * to reveal the spatial clustering and differentiation characteristics of KES. The Getis-Ord Gi* statistic is a robust method for identifying statistically significant spatial clusters of high values (hotspots) and low values (cold spots)^54^. The calculation formula is as follows:

1 $$\:{G}_{i}^{\mathrm{*}}=\frac{{\sum\:}_{j=1}^{n}{{w}_{i}}_{j}\times\:{x}_{j}-\overline{X}{\sum\:}_{j=1}^{n}{{w}_{i}}_{j}}{S\sqrt{\frac{n{\sum\:}_{j=1}^{n}{{{w}_{i}}_{j}}^{2}-{\left({\sum\:}_{j=1}^{n}{{w}_{i}}_{j}\right)}^{2}}{n-1}}}$$

2 $$\:\overline{X}=\frac{{\sum\:}_{j=1}^{n}{x}_{j}}{n}$$

3 $$\:S=\sqrt{\frac{{\sum\:}_{j=1}^{n}{{x}_{j}}^{2}}{n-1}-{\left(\overline{X}\right)}^{2}}$$

Where $\:{X}_{j}$ is the attribute for the county, $\:\stackrel{-}{X}$ is the global mean, S is the standard deviation of x, and $\:{W}_{ij}$ is the spatial weight.

### Relative change rate index

This study utilizes the relative change rate index to quantitatively characterize the dynamic evolutionary trends of three KES from 2015 to 2020. The calculation formula is as follows:

4 $$\:{V}_{\mathrm{ij}}=\frac{{B}_{ij}-{A}_{ij}}{{A}_{ij}}\times\:100\mathrm{\%}$$

Where $\:{V}_{ij}$ represents the relative change rate of the total volume for KES category j in county i; $\:{B}_{ij}$ and $\:{A}_{ij}$ denote the total quantities of the j-th KES in county $\:i$ for the years 2020 and 2015, respectively. A positive $\:{V}_{ij}$ signifies functional enhancement, whereas a negative value indicates functional degradation. The classification of relative change rates in this study was established by integrating the natural fluctuation dynamics of ecosystem services, inherent model uncertainties, and established conventions in macro-ecological assessments. Given that ecosystem services are highly sensitive to minor interannual variations in meteorological drivers (e.g., precipitation and wind speed), and that remote sensing inputs and empirical assessment models inevitably introduce systematic errors, defining fluctuations within a ± 5% margin as natural variability and assessment errors is methodologically necessary. This threshold effectively prevents temporary climatic anomalies from being misinterpreted as structural ecological degradation or improvement^55^. Furthermore, drawing upon quartile-based statistical distributions and standardized land degradation assessment frameworks, variations exceeding ± 25% generally denote clearly observable, moderate shifts in ecosystem structure or function. Conversely, variations exceeding ± 50% signify severe, drastic, or even fundamental state alterations^56^. Accordingly, the relative change rate index was stratified into the following seven categories based on the severity of the change: severe decline ($\:{V}_{ij}$ < -50%), moderate decline (-50% ≤ $\:{V}_{ij}$ < -25%), light decline (-25% ≤ $\:{V}_{ij}$ < -5%), stable (-5% ≤ $\:{V}_{ij}$ < 5%), light increase (5% ≤ $\:{V}_{ij}$ < 25%), moderate increase (25% ≤ $\:{V}_{ij}$ < 50%), and severe increase ($\:{V}_{ij}$ ≥ 50%).

### Construction of ecosystem services early-warning model

**Fig. 3:**
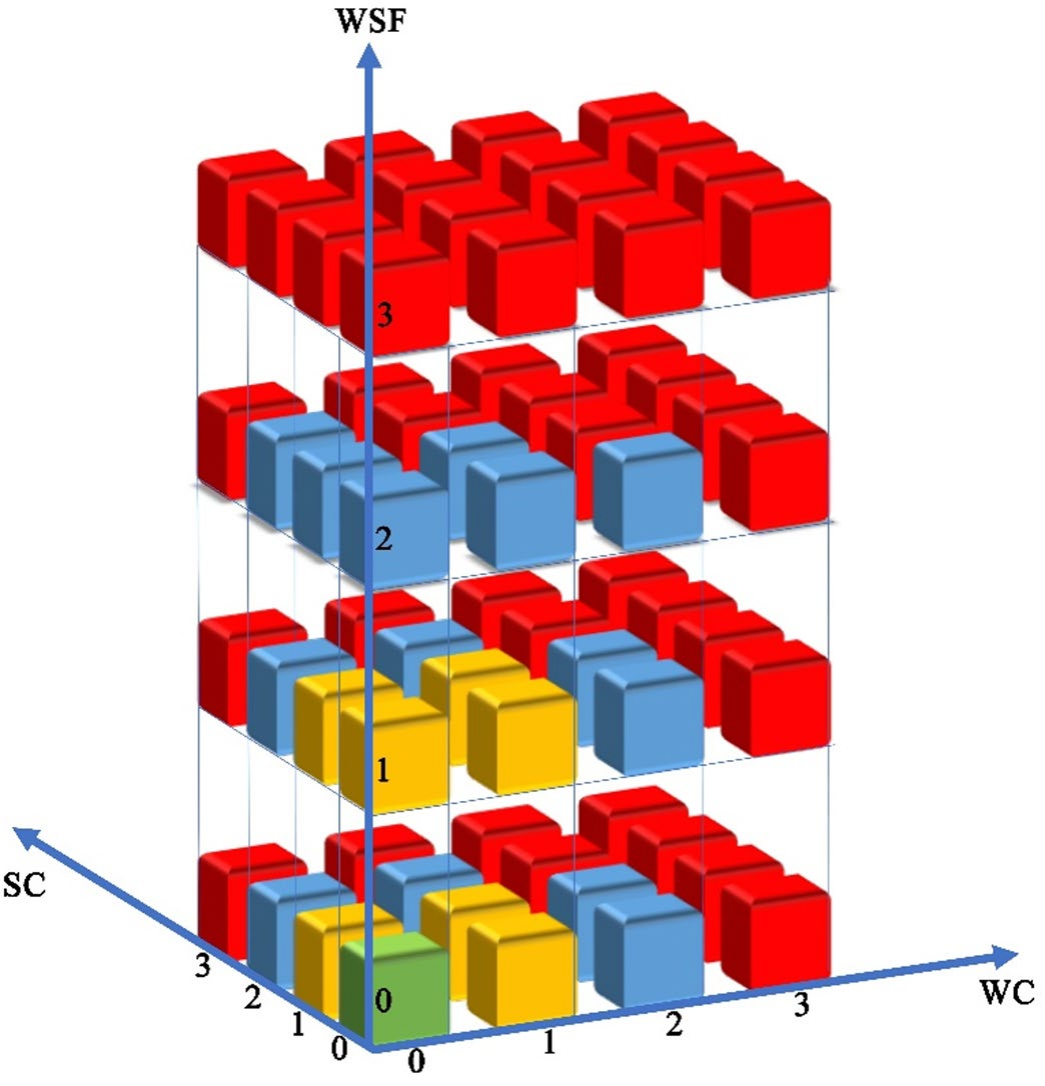
Early-warning model for ecosystem services.

To identify the degraded areas and their corresponding intensities, this study utilized 2,847 county-level administrative divisions as the fundamental assessment units. The relative change rates for WC, SC, WSF were categorized into four degradation levels: Level 0 (stable or increasing), Level 1 (light decline), Level 2 (moderate decline), and Level 3 (severe decline). A three-dimensional coding system (WC_x_, SC_x_, WSF_x_) was developed to characterize the integrated degradation structure of the three KES at the county scale, where x denotes the degradation level (0–3) of each service. Based on this system, a KES degradation early-warning model (Fig. 3) and an alert discrimination matrix comprising 64 combinatorial states (Table 1) were established. Alert intensities are classified into four tiers: no alert (green), light alert (yellow), moderate alert (blue), and severe alert (red). The specific determination rules are as follows: No alert is assigned when the degradation levels of all three services are Level 0; Light alert is triggered when only one or two services are at Level 1; Moderate alert is designated if any single service reaches Level 2; Severe alert is issued if any service reaches Level 3, or if two or more services simultaneously reach Level 2.

**Table 1:** Ecosystem service degradation early-warning matrix.

Degradation level combination	Alert level	Degradation level combination	Alert level	Degradation level combination	Alert level
(WC_0_, SC_0_, WSF_0_)	No alert	(WC_3_, SC_0_, WSF_0_)	Severe alert	(WC_1_, SC_3_, WSF_1_)	Severe alert
(WC_1_, SC_0_, WSF_0_)	Light alert	(WC_2_, SC_2_, WSF_0_)	Severe alert	(WC_2_, SC_1_, WSF_2_)	Severe alert
(WC_0_, SC_1_, WSF_0_)	Light alert	(WC_0_, SC_2_, WSF_2_)	Severe alert	(WC_2_, SC_2_, WSF_1_)	Severe alert
(WC_0_, SC_0_, WSF_1_)	Light alert	(WC_2_, SC_0_, WSF_2_)	Severe alert	(WC_3_, SC_1_, WSF_1_)	Severe alert
(WC_1_, SC_1_, WSF_0_)	Light alert	(WC_1_, SC_0_, WSF_3_)	Severe alert	(WC_1_, SC_2_, WSF_3_)	Severe alert
(WC_1_, SC_0_, WSF_1_)	Light alert	(WC_1_, SC_3_, WSF_0_)	Severe alert	(WC_1_, SC_3_, WSF_2_)	Severe alert
(WC_0_, SC_1_, WSF_1_)	Light alert	(WC_3_, SC_1_, WSF_0_)	Severe alert	(WC_2_, SC_1_, WSF_3_)	Severe alert
(WC_1_, SC_1_, WSF_1_)	Light alert	(WC_3_, SC_0_, WSF_1_)	Severe alert	(WC_2_, SC_2_, WSF_2_)	Severe alert
(WC_0_, SC_0_, WSF_2_)	Moderate alert	(WC_0_, SC_3_, WSF_1_)	Severe alert	(WC_2_, SC_3_, WSF_1_)	Severe alert
(WC_0_, SC_2_, WSF_0_)	Moderate alert	(WC_0_, SC_1_, WSF_3_)	Severe alert	(WC_3_, SC_1_, WSF_2_)	Severe alert
(WC_2_, SC_0_, WSF_0_)	Moderate alert	(WC_0_, SC_2_, WSF_3_)	Severe alert	(WC_3_, SC_2_, WSF_1_)	Severe alert
(WC_0_, SC_2_, WSF_1_)	Moderate alert	(WC_0_, SC_3_, WSF_2_)	Severe alert	(WC_1_, SC_3_, WSF_3_)	Severe alert
(WC_2_, SC_1_, WSF_0_)	Moderate alert	(WC_2_, SC_3_, WSF_0_)	Severe alert	(WC_2_, SC_2_, WSF_3_)	Severe alert
(WC_2_, SC_0_, WSF_1_)	Moderate alert	(WC_3_, SC_0_, WSF_2_)	Severe alert	(WC_2_, SC_3_, WSF_2_)	Severe alert
(WC_0_, SC_1_, WSF_2_)	Moderate alert	(WC_2_, SC_0_, WSF_3_)	Severe alert	(WC_3_, SC_1_, WSF_3_)	Severe alert
(WC_1_, SC_2_, WSF_0_)	Moderate alert	(WC_3_, SC_2_, WSF_0_)	Severe alert	(WC_3_, SC_2_, WSF_2_)	Severe alert
(WC_1_, SC_0_, WSF_2_)	Moderate alert	(WC_0_, SC_3_, WSF_3_)	Severe alert	(WC_3_, SC_3_, WSF_1_)	Severe alert
(WC_1_, SC_2_, WSF_1_)	Moderate alert	(WC_3_, SC_0_, WSF_3_)	Severe alert	(WC_2_, SC_3_, WSF_3_)	Severe alert
(WC_2_, SC_1_, WSF_1_)	Moderate alert	(WC_3_, SC_3_, WSF_0_)	Severe alert	(WC_3_, SC_2_, WSF_3_)	Severe alert
(WC_1_, SC_1_, WSF_2_)	Moderate alert	(WC_1_, SC_1_, WSF_3_)	Severe alert	(WC_3_, SC_3_, WSF_2_)	Severe alert
(WC_0_, SC_0_, WSF_3_)	Severe alert	(WC_1_, SC_2_, WSF_2_)	Severe alert	(WC_3_, SC_3_, WSF_3_)	Severe alert
(WC_0_, SC_3_, WSF_0_)	Severe alert				

## Results

### Overall trends of KES in China

**Fig. 4:**
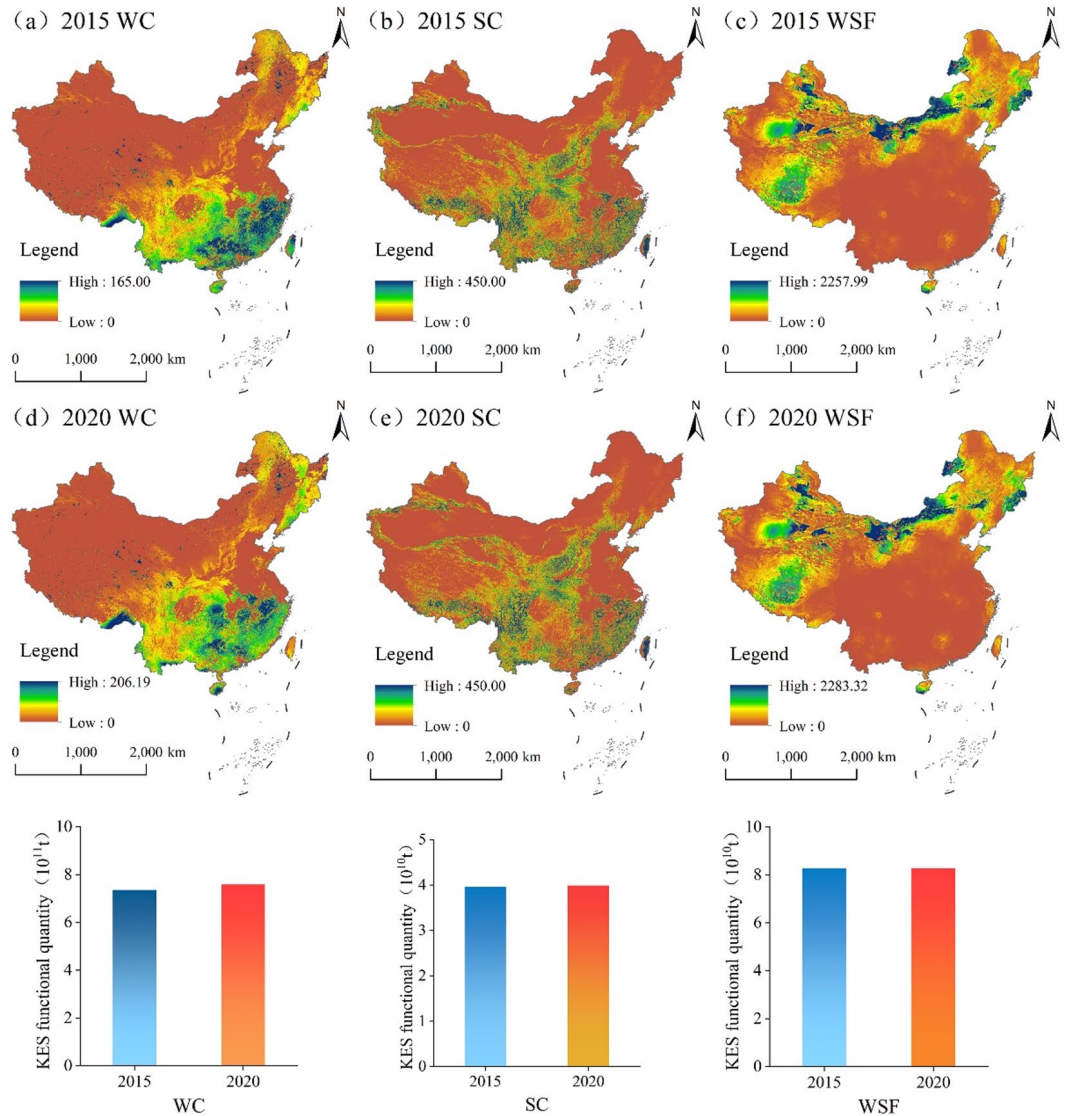
Spatial pattern and trends in the KES in China: 2015 and 2020. (The map was generated using ArcGIS 10.8 and Origin 2020).

As shown in Fig. 4, the aggregated KES in China exhibited a generally stable trend with a slight increase from 2015 to 2020. Specifically, WC showed a distinct increase of 3.13%, rising from 7.36 × 10¹¹ t to 7.59 × 10¹¹ t. In comparison, SC and WSF remained relatively stable, showing marginal growth rates of 0.51% (from 3.96 × 10¹⁰ t to 3.98 × 10¹⁰ t) and 0.12% (from 8.26 × 10¹⁰ t to 8.27 × 10¹⁰ t), These findings suggest that China’s ecological protection and restoration measures have effectively maintained and enhanced KES during the monitoring period.

### Spatiotemporal patterns of KES in China

#### Spatiotemporal patterns of WC

**Fig. 5:**
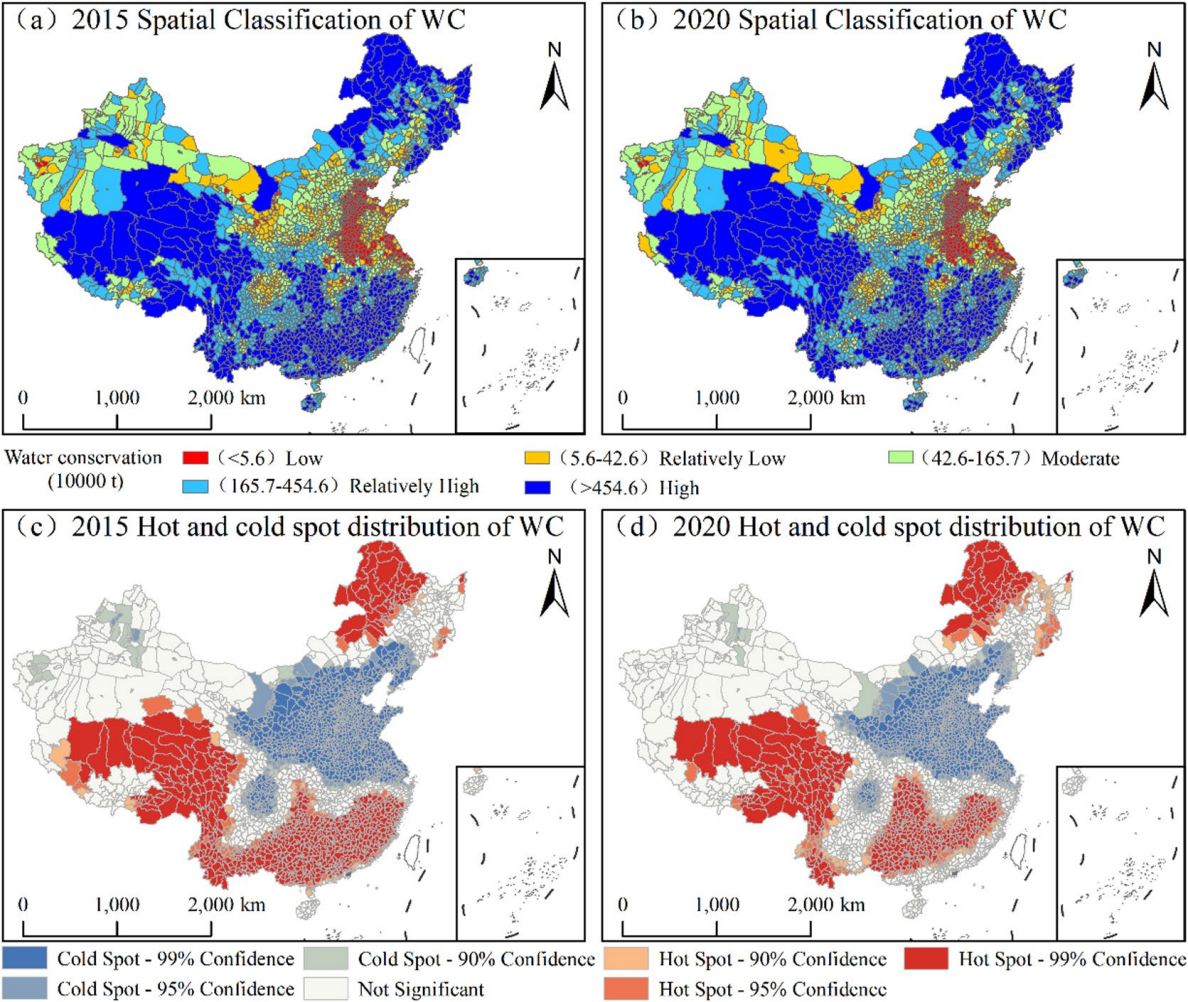
Spatiotemporal patterns and cold-hot spot distribution of WC in China, 2015–2020. (The map was generated using ArcGIS 10.8).

During the monitoring period, the spatial distribution of WC at the county level in China was characterized by the fragmented clustering of high-value areas and the contiguous distribution of low-value zones. The overall configuration of hotspots and cold spots remained stable, with localized optimizations (Fig. 5). High-value regions were predominantly concentrated in the QTP, southern mountainous areas, and key forest zones in Northeast China, which collectively constitute the core ecological barrier for national WC. By 2020, the number of high-value counties reached 561, a slight decrease from 2015; however, their cumulative functional volume was approximately 5.1 × 10^11^ t, accounting for 68% of the national total. Meanwhile, the number of counties in moderate-value and relatively high-value zones increased from 557 to 582 and from 550 to 590, respectively. These findings corroborate the aforementioned conclusion that China’s WC capacity is following a stable-to-increasing trajectory.

### Spatiotemporal patterns of SC

**Fig. 6:**
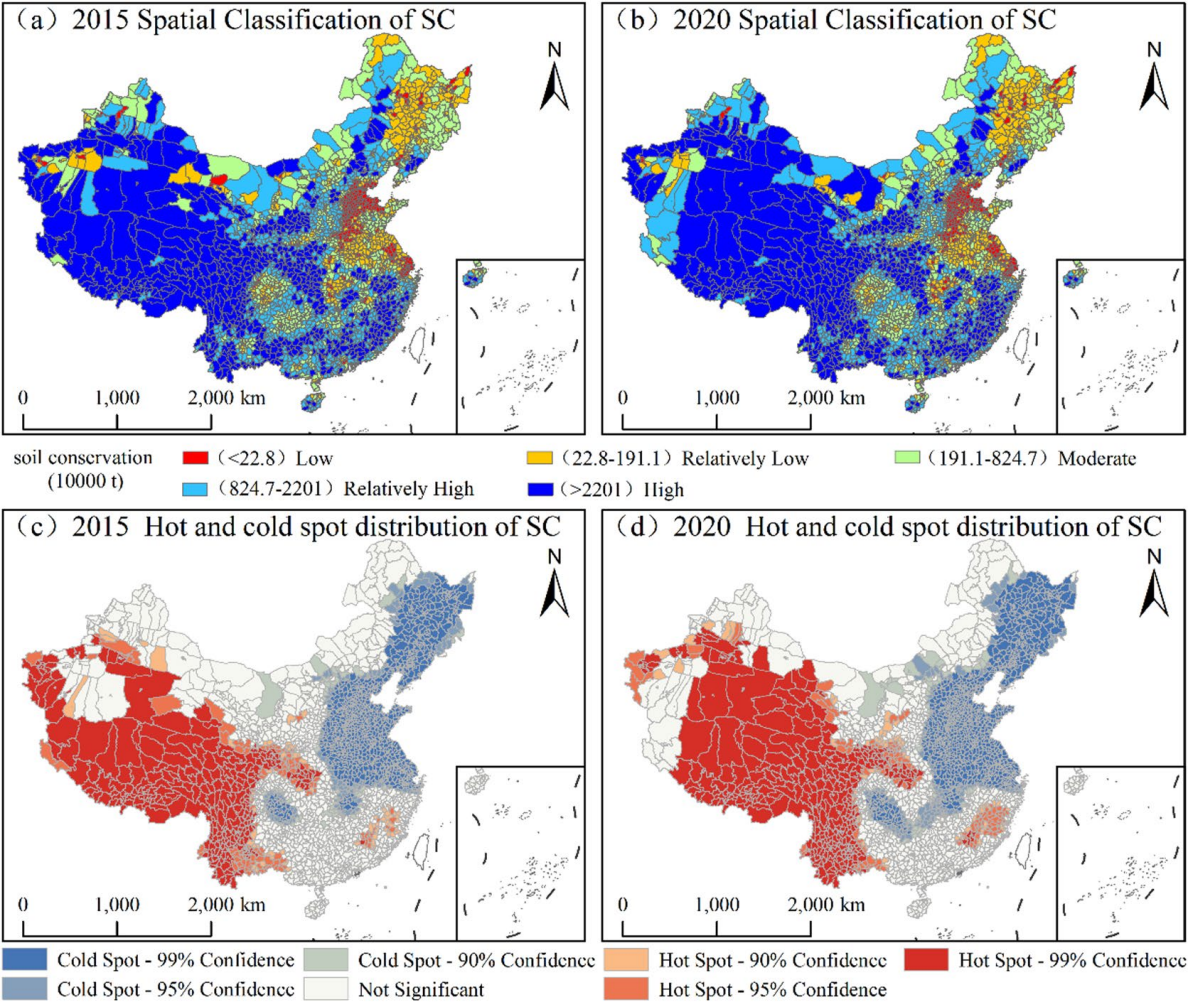
Spatiotemporal patterns and cold-hot spot distribution of SC in China, 2015–2020. (The map was generated using ArcGIS 10.8).

During the monitoring period, the county-level SC service in China exhibited a pronounced “high-in-the-southwest, low-in-the-northeast” spatial distribution. The extent of hotspot regions expanded slightly, while the distribution of cold-spots remained relatively stable (Fig. 6). High-value zones were primarily concentrated in key ecological regions, including the Loess Plateau, QTP, Yunnan-Guizhou Plateau, and the hilly regions of Southeast China, which collectively constitute the core functional areas for national SC. By 2020, the number of high-value counties reached 572, with a cumulative functional volume of approximately 2.8 × 10¹⁰ t, accounting for 72% of the national total—a slight increase compared to 2015. The stability observed in the number of counties within medium- and low-value zones further indicates that China’s SC capacity remained overall consistent, with no significant fluctuations in the macro-regional configuration.

### Spatiotemporal patterns of WSF

**Fig. 7:**
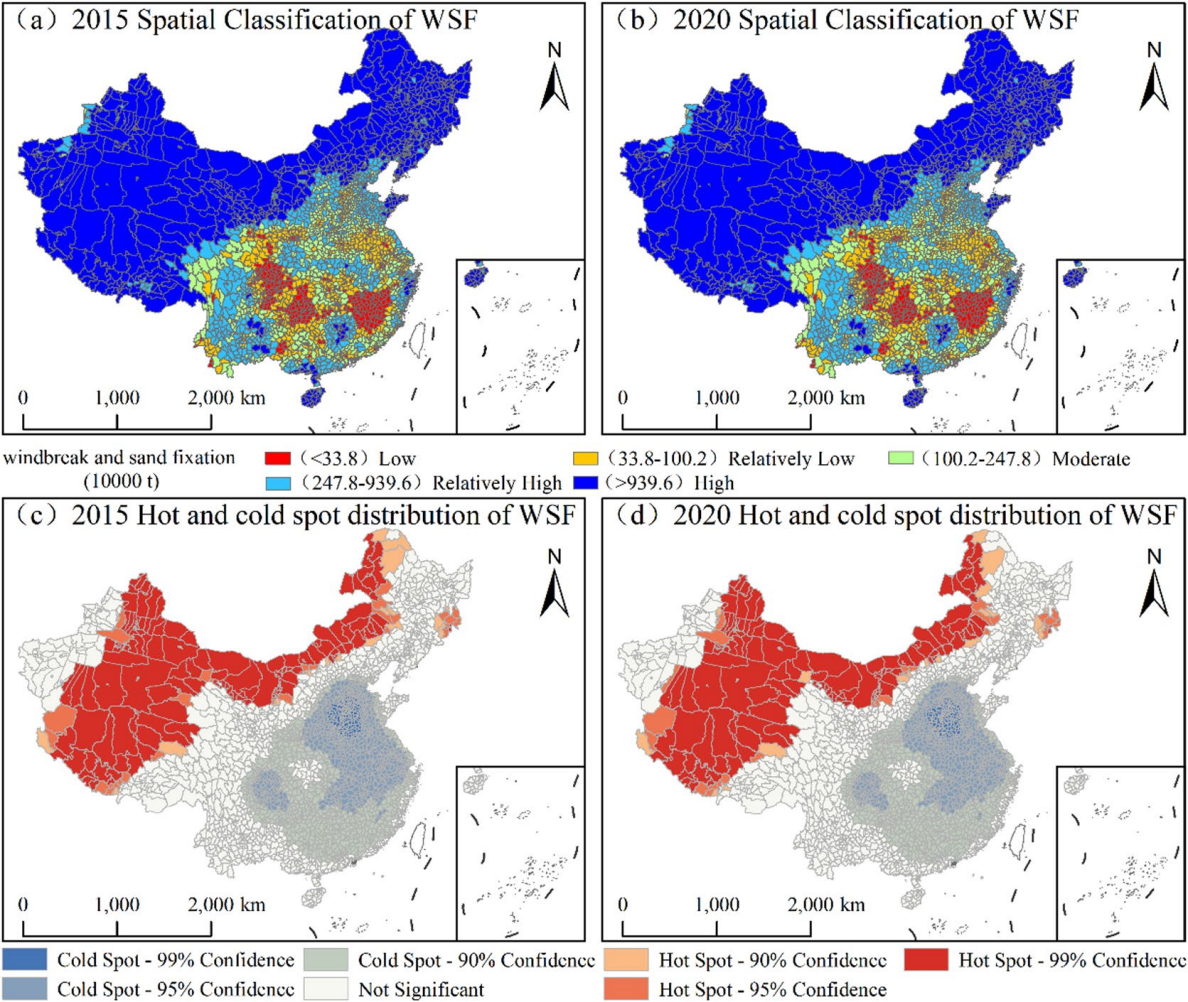
Spatiotemporal patterns and cold-hot spot distribution of WSF in China, 2015–2020. (The map was generated using ArcGIS 10.8).

During the monitoring period, the spatial pattern of WSF at the county level in China exhibited a distinct “high-in-the-northwest, low-in-the-southeast” distribution, with significant spatial clustering of hotspots and cold-spots (Fig. 7). High-value zones were highly concentrated in the northern arid and semi-arid regions and the QTP, which serve as the core support areas for national WSF services. By 2020, the number of high-value counties was 570, with a cumulative functional volume of 7.9 × 10¹⁰ t. This accounted for 95% of the national total, underscoring the decisive role of these high-value zones in sustaining this ecosystem service. Meanwhile, the number of counties in moderate-value zones decreased slightly, whereas those in low and relatively low-value zones remained stable.

### Evolution characteristics of KES in China

#### Evolution characteristics of WC

**Fig. 8:**
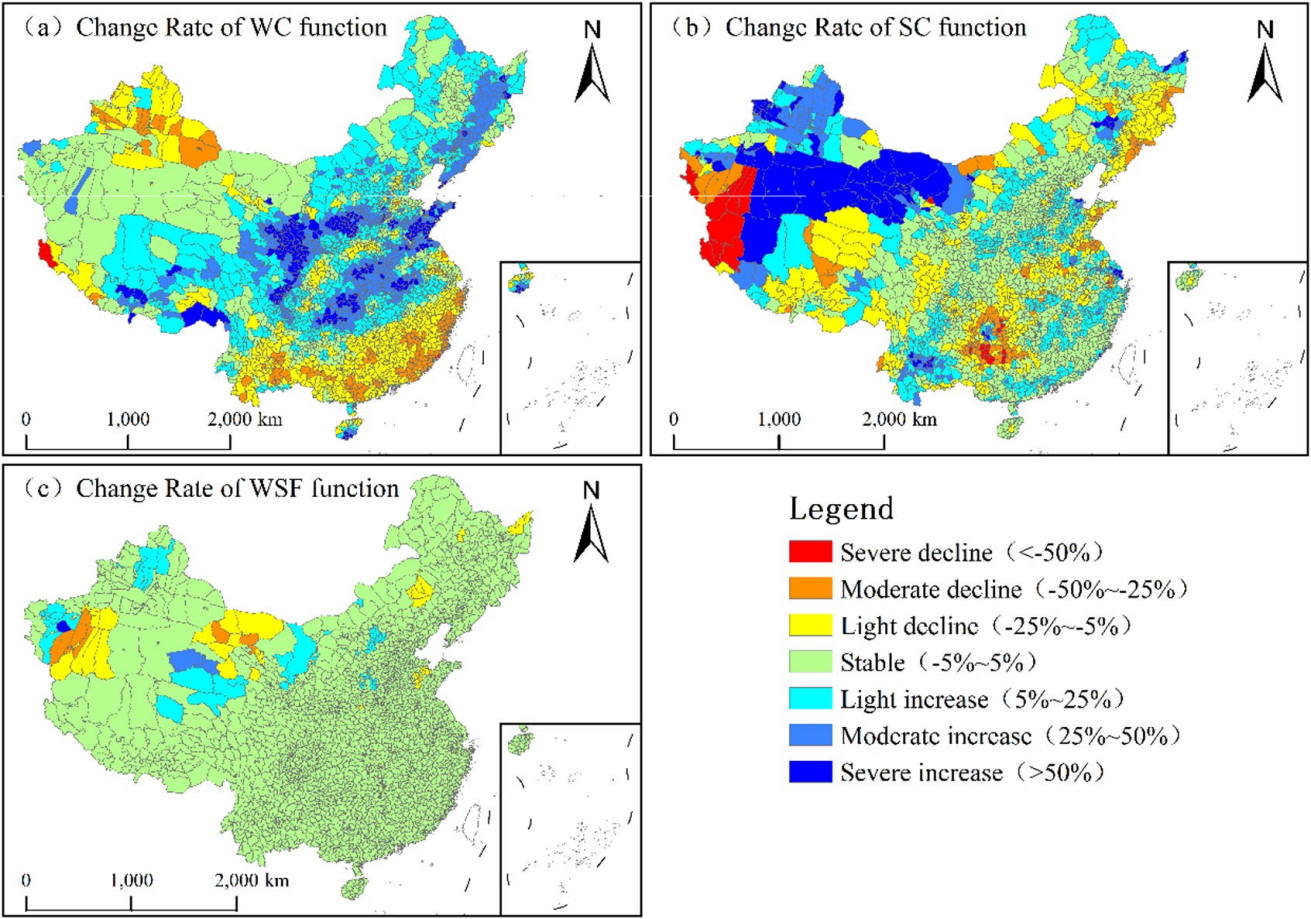
Spatial distribution of evolution types of KES in China, 2015–2020. (**a**) Change rate of WC function. (**b**) Change rate of SC function. (**c**) Change rate of WSF. (The map was generated using ArcGIS 10.8).

During the monitoring period, the dynamic evolution of WC across China’s counties exhibited distinct spatial heterogeneity, characterized by a pattern of “predominant growth, secondary degradation, and supplementary stability” (Fig. 8a; Table 2). Growth-type counties numbered 1515 (53.21%), predominantly exhibiting mild to moderate increases. These counties were spatially concentrated in key ecological zones, including Sanjiangyuan, the Gannan Grasslands, the Changbai Mountains, the Qinling-Bashan Mountains, the Taihang Mountains, the Wuling-Dabie Mountains, the Shandong Hills, and the Loess Plateau, indicating a significant enhancement in WC services within these regions. In contrast, 734 counties (25.78%) experienced degradation, dominated by slight declines. These areas were mainly located in South China, the Yunnan-Guizhou Plateau, and Northern Xinjiang, identifying them as priority zones for future ecological restoration and functional enhancement. Finally, 589 counties (20.69%) remained stable.

### Evolution characteristics of SC

During the monitoring period, the evolution of SC capacity across China’s counties exhibited a spatial pattern characterized as “predominantly stable, supplemented by growth, with coexisting degradation” (Fig. 8b; Table 2). Stable counties constituted the largest group, totaling 1,174 (41.24%). A total of 941 counties (33.05%) showed an increasing trend, primarily consisting of slight growth. These were mainly distributed across Xinjiang, northwestern Gansu, western Inner Mongolia, the Loess Plateau, Yunnan, and the hilly regions of Southern China, reflecting the effective maintenance and enhancement of SC services in these areas. In contrast, 732 counties (25.71%) experienced degradation, also dominated by slight declines. These were concentrated in Guizhou, southern Xinjiang, eastern Tibet (Xizang), and the Changbai Mountains, identifying them as priority regions for future soil erosion control and ecological restoration.

#### Evolution characteristics of WSF

During the monitoring period, the evolution of WSF across China’s counties exhibited a spatial pattern characterized as “absolute stability supplemented by minor fluctuations” (Fig. 8c; Table 2). Stable counties reached a total of 2,708, accounting for 95.12% of the national total. Counties experiencing change were exceptionally rare: only 80 counties exhibited an increasing trend, primarily characterized by slight growth and scattered across Kashgar and Changji in Xinjiang, eastern Qinghai, and localized areas of Inner Mongolia. Conversely, 59 counties showed degradation, dominated by slight declines mainly occurring in Hotan (Xinjiang) and northwestern Gansu. These findings indicate that China’s WSF services remained exceptionally stable overall, with significant changes confined to highly localized areas within the northwest region.

**Table 2:** Statistics on the number of counties for different evolution types of KES in China, 2015–2020.

KES	Evolution types						
	Severe decline	Moderate decline	Light decline	Stable	Light increase	Moderate increase	Severe increase
WC	10	182	551	589	772	489	254
SC	41	164	527	1174	724	125	92
WSF	0	8	51	2708	77	2	1

#### Analysis of degradation early-warning for KES in China

**Fig. 9:**
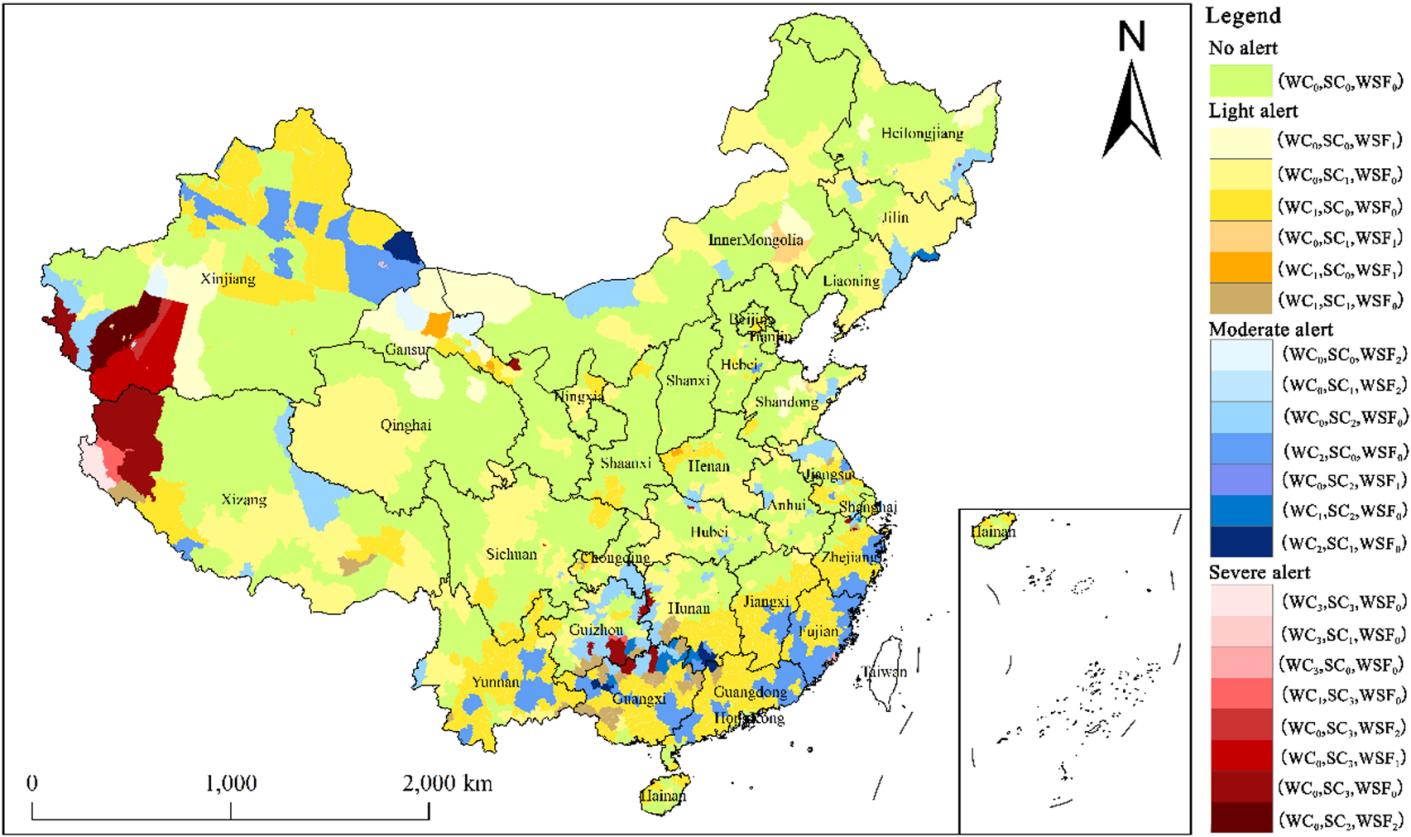
Spatial distribution of degradation early-warning for KES in China. (The map was generated using ArcGIS 10.8).

Utilizing the KES degradation early-warning model (Fig. 3) and the alert discrimination matrix (Table 1), this study identified four alert intensities of KES degradation across China from 2015 to 2020 (Table 3), along with their corresponding spatial distributions (Fig. 9). Overall, the degradation alerts were predominantly categorized as “no alert” and “light alert,” while moderate and severe alerts accounted for a relatively small proportion of the assessment units.

A total of 1,394 counties were classified as “no alert” (green), accounting for 48.96% of the total. This finding indicates that nearly half of China’s counties maintained stable or improving KES trends during the monitoring period.

A total of 1,052 counties were classified as “light alert” (yellow), accounting for 36.97% of the assessment units. This status suggests that these regions experienced slight degradation in one or two functions, necessitating their integration into routine monitoring systems. Light alerts primarily stemmed from the slight decline in WC and SC services. Specifically, the degradation structures (WC_1_, SC_0_, WSF_0_) and (WC_0_, SC_1_, WSF_0_) accounted for 46.67% and 44.87% of this category, respectively. Spatially, light-alert counties driven by WC degradation were concentrated in Guangdong, Jiangxi, Fujian, Zhejiang, Hunan, Guangxi, Yunnan, and northern Xinjiang. Conversely, those dominated by SC degradation were primarily distributed across Qinghai, Tibet, Sichuan, Jilin, Liaoning, and Henan.

A total of 349 counties were classified as “moderate alert” (blue), accounting for 12.26% of the total. This status reflects that at least one ecosystem service in these regions has undergone moderate degradation, necessitating their designation as priority monitoring targets. Similar to light alerts, moderate alerts primarily originated from the moderate decline in WC and SC services. Specifically, the degradation structures (W_2_, S_0_, F_0_) and (W_0_, S_2_, F_0_) accounted for 50.14% and 42.69% of this category, respectively. Geographically, moderate-alert counties driven by WC degradation were mostly distributed in Guangdong, Fujian, Zhejiang, Jiangxi, Guangxi, Yunnan, and northern Xinjiang. Conversely, those dominated by SC degradation were concentrated in Guizhou, southeastern Chongqing, northern Jiangsu, southern Jilin, eastern Heilongjiang, and eastern Tibet.

A total of 52 counties were identified as “severe alert” (red), accounting for a mere 1.83% of the total. This alert level was primarily driven by the substantial degradation of SC services, which accounted for 63.46% of the severe-alert cases. Geographically, these counties are concentrated in Hotan (Xinjiang), Ngari (Tibet), and the bordering areas of Guizhou, Guangxi, and Hunan. These regions are characterized by either the severe decline of a single function or the cumulative effect of moderate decline across multiple functions. Given the pronounced ecosystem risks in these areas, emergency restoration and regulatory interventions are urgently required.

**Table 3:** Statistics of early-warning levels and county numbers of KES in China.

Alert level	Degradation level combination	Number of counties
No alert (Green)	(WC_0_, SC_0_, WSF_0_)	1394
Light alert (Yellow)	(WC_0_, SC_0_, WSF_1_)	40
	(WC_0_, SC_1_, WSF_0_)	472
	(WC_1_, SC_0_, WSF_0_)	491
	(WC_1_, SC_0_, WSF_1_)	3
	(WC_1_, SC_1_, WSF_0_)	42
	(WC_0_, SC_1_, WSF_1_)	4
Moderate alert (Blue)	(WC_0_, SC_0_, WSF_2_)	4
	(WC_2_, SC_0_, WSF_0_)	175
	(WC_0_, SC_2_, WSF_0_)	149
	(WC_1_, SC_2_, WSF_0_)	12
	(WC_0_, SC_2_, WSF_1_)	1
	(WC_0_, SC_1_, WSF_2_)	1
	(WC_2_, SC_1_, WSF_0_)	7
Severe alert (Red)	(WC_0_, SC_2_, WSF_2_)	2
	(WC_0_, SC_3_, WSF_0_)	33
	(WC_0_, SC_3_, WSF_1_)	3
	(WC_0_, SC_3_, WSF_2_)	1
	(WC_1_, SC_3_, WSF_0_)	3
	(WC_3_, SC_0_, WSF_0_)	8
	(WC_3_, SC_1_, WSF_0_)	1
	(WC_3_, SC_3_, WSF_0_)	1

## Discussion

### Comparison between this study and previous studies

Focusing on the critical period of China’s ecological civilization construction from 2015 to 2020, this study developed a change assessment and degradation early-warning framework for KES at the county scale. This framework systematically reveals the spatiotemporal differentiation of three vital KES—WC, SC, and WSF—and enables the precise identification of spatial patterns in KES degradation alerts.

Findings indicate that between 2015 and 2020, the three KES components nationwide maintained an overall trend of “stable with slight increases.” This evolution aligns with existing research on changes in key ecosystem service functions^17–19^. This partially reflects the initial effectiveness of ecological conservation and restoration policies under sustainable development goals since ecological civilization construction was elevated to a national strategy in China. However, ecosystem service degradation persists in some counties, indicating that regional policy impacts remain limited in certain areas^46^. These changes in KES across counties underscore the value of conducting change assessment studies during the critical policy period from 2015 to 2020.

Regarding spatial patterns, previous studies have examined WC^14^, SC^15^, and WSF^17^ across diverse administrative and grid scales, albeit predominantly focusing on localized regions. In contrast, this study systematically elucidates the national distribution of high-value zones for these three KES at the county scale. Specifically, high-value WC areas are concentrated in the QTP, southern mountainous regions, and key forest zones of Northeast China, aligning closely with the National Key Ecological Functional Zones for WC^31,57^. High-value SC regions are distributed across the Loess Plateau, QTP, Yunnan-Guizhou Plateau, and the hilly regions of Southeast China, which constitute the mainstay of the “Loess Plateau-Sichuan-Yunnan Ecological Barrier"^19^. Furthermore, high-value WSF zones are localized within the northern arid and semi-arid regions and the QTP, encompassing critical areas such as the Three-North Shelter Forest Program and the Northern Sand Prevention Belt^17,58^.

Regarding the methodological framework for analyzing the spatiotemporal changes of ecosystem services at the county level, this study adopts the total functional quantity rather than the per-unit-area mean as the primary evaluation metric. This approach diverges from conventional pure ecological assessments, which routinely employ mean-value methods to eliminate the area effect^35,36^. We argue that within a spatial governance system anchored by administrative divisions, the evaluation perspective must transition from assessing ‘pure ecological efficiency’ to serving macro-administrative management and policy implementation. First, total quantity indicators provide a more objective reflection of a region’s absolute contribution to national ecological security. Due to climatic constraints, mega-counties in western China and macro-ecological zones like the Qinghai-Tibet Plateau generally exhibit low ecological function intensity per unit area. However, owing to their vast spatial expanses, their absolute ecological contributions remain irreplaceable^19,31^. Adopting a mean-based approach would likely mask this critical ‘area effect’, thereby severely underestimating the strategic value of these regions as national ecological barriers. Furthermore, utilizing total physical quantities and assessing their relative changes provides a tangible baseline for the subsequent early-warning model, enabling a more intuitive and direct representation of actual ecological risks.

Regarding the methodology for ecological degradation early warning, this study diverges significantly from established paradigms. Previous research predominantly identifies risks by coupling multidimensional indicators into composite indices^47–49^ or by projecting future potential risks through scenario simulations^51–53^. Although multidimensional composite indices provide a holistic view of regional ecological health, significant degradation in a single key ecosystem service can be statistically masked by improvements in others, thereby obscuring underlying structural ecological imbalances^50^. While scenario modeling is highly advantageous for long-term strategic planning and forecasting degradation risks across various socioeconomic pathways, such predictive frameworks are inherently constrained by high methodological uncertainties, which can compromise both their accuracy and interpretability^53^. In contrast, the framework developed in this study relies directly on the Relative Change Rate of three KES categories over the recent period (2015–2020). By quantifying relative degradation levels and implementing a three-dimensional coding system, this approach ensures the transparent transmission of KES change signals, offering an intuitive tool for managers to rapidly localize problematic regions. This “fact-based” early-warning approach circumvents the uncertainties inherent in complex scenario simulations and delivers direct evidence of functional shifts. Characterized by procedural clarity, computational simplicity, and cross-scale applicability, this framework serves as a valuable complement to existing research methods.

### Policy recommendations based on the degradation early-warning of KES in China

This study represents the first systematic identification of four-level degradation alerts for three KES at the national county scale (Fig. 9; Table 3). The results indicate that nearly half of the counties (48.96%) are categorized as “no alert,” reflecting the overall positive progress of China’s ecological civilization construction. However, more than half of the counties exhibit varying degrees of functional degradation, characterized by significant spatial heterogeneity in alert patterns. This differentiation is associated with a complex interplay of factors, including natural environmental baselines, anthropogenic activities, and climate change^10,32,38^. Consequently, the following tiered governance policy recommendations are proposed, tailored to specific alert types and priority regions.

Governance in “No-alert” Counties (1,394) should focus on consolidating existing ecological functions. Successful models of ecological conservation and restoration should be systematically synthesized and promoted, and subsequently integrated into the standardized assessment frameworks for local ecological civilization development. And strategic efforts should progressively pivot toward the value realization of ecological assets by actively exploring and establishing cross-regional horizontal eco-compensation mechanisms. Furthermore, a dynamic monitoring mechanism must be established to mitigate potential risks associated with climate change and future land-use transitions, thereby ensuring the long-term stability and resilience of ecosystem services.

The “Light-alert” Counties (1,052) serves as a critical buffer zone to prevent the spread of ecological degradation. The alerts are primarily driven by the slight degradation of WC or SC services. Governance in these areas should adhere to the principle of “early detection and timely intervention,” with a focus on strengthening routine monitoring to prevent progression toward moderate degradation. It is recommended to eschew large-scale anthropogenic interventions in favor of decentralized ecological maintenance measures. Site-specific strategies—such as the targeted remediation of sloping croplands, the conservation of micro-watersheds, and the silvicultural tending of shelterbelts—should be prioritized to facilitate the natural recovery of ecological functions^45^.

The “Moderate-alert” Counties (349) represent the primary frontline for ecological governance, as their alerts are largely driven by the moderate degradation of WC or SC services. Within these areas, the Ecological Protection Red Line system must be strictly enforced to restrict anthropogenic activities, while moderately promoting low-impact green industries such as ecotourism and ecological agriculture^59^. Concurrently, it is imperative to implement targeted and systematic ecological engineering projects, including comprehensive small-watershed management, afforestation, and degraded grassland rehabilitation. Furthermore, ecological recovery outcomes must be integrated into binding performance metrics for local governments. Such rigid institutional constraints will ensure the effective on-the-ground implementation of restoration efforts, thereby driving the trajectory of ecological functions from moderate degradation back to a stable state^60,61^.

The “Severe-alert” Counties (52) represent the most critical vulnerabilities in national ecological security, with alerts primarily driven by the substantial degradation of SC services. These areas are concentrated in the extreme arid zones of Northwest China (e.g., Hotan in Xinjiang and Ngari in Tibet) and the karst mountainous regions of Southwest China (e.g., the border areas of Guizhou, Guangxi, and Hunan), where human-land conflicts are acute. Consequently, the most stringent conservation and restoration measures are mandatory. Given the extreme ecological fragility and severe fiscal constraints inherent to these regions, local governments are fundamentally ill-equipped to independently reverse systemic degradation. Consequently, governance frameworks must synergize stringent development restrictions with national-level coordination. On the one hand, in extreme arid zones such as Hotan and Ngari, priority must be given to deploying water-saving and sand-stabilization technologies and constructing windbreak-sand-fixation belts, combined with strict grazing bans, fallowing, and ecological water replenishment to facilitate surface recovery^17,20^. For the southwestern karst regions, interventions should focus on rocky desertification control through a combination of engineering measures (e.g., slope-to-terrace conversion, small-scale water conservancy, and soil amelioration) and biological measures (e.g., “forest-shrub-grass” complex restoration systems). Furthermore, ecological resettlement and industrial transformation should be advanced to mitigate anthropogenic disturbance^62^. On the other hand, local authorities should leverage the absolute quantitative changes in KES and their associated risk profiles—as quantified in this study—as an objective baseline to actively secure national special funds for desertification and rocky desertification mitigation, alongside vertical ecological transfer payments. This robust infusion of external capital is indispensable for underwriting the natural recovery and long-term rehabilitation of these vulnerable ecosystems.

## Limitations and prospects

The limitations of this study are primarily reflected in two aspects. First, the time span of dynamic monitoring is relatively restricted. Although focusing on the critical period of 2015–2020 captures the initial impact of China’s ecological civilization construction, it remains insufficient to fully elucidate the long-term evolutionary patterns of ecosystem services or the enduring effects of national strategies. Subsequent research should establish longer time series, organically linking the pre-implementation phase (2000–2015), initial implementation phase (2015–2020), and subsequent consolidation phase (post-2020) to systematically evaluate the dynamic evolution of China’s KES and the sustained effects of ecological governance. Second, the attribution analysis of degradation alerts remains to be deepened. While the developed early-warning framework effectively identifies spatial disparities in alert intensities, it has yet to quantitatively decipher the underlying driving mechanisms. Future studies should integrate multi-source datasets—encompassing climate change, natural environmental baselines, and anthropogenic activities—and employ statistical attribution methods to distinguish the relative contributions of natural versus human factors. This would complete the comprehensive “assessment–early warning–attribution–governance” research framework, providing more robust scientific support for the ecological management of national territorial space. Finally, given China’s vast territorial expanse, significant spatial heterogeneity exists in natural baselines, climatic conditions, and ecosystem resilience across different regions. Future research should comprehensively integrate regional eco-geographical characteristics and environmental carrying capacities to develop context-specific, differentiated, and dynamic threshold systems. Doing so will further enhance the precision and applicability of the early-warning model within complex geographical environments.

## Conclusions

This study focuses on the pivotal phase of China’s ecological civilization construction from 2015 to 2020, this study represents the first attempt to systematically develop a framework for monitoring spatiotemporal variations and early warning of degradation for three KES—WC, SC, and WSF—at the national county scale. This framework elucidates the spatiotemporal changes of these services and identifies their corresponding degradation alert intensities. The principal conclusions are summarized as follows:

1. During the monitoring period, the aggregate capacity of China’s three KES exhibited an overall trend of “stability with slight growth.” Specifically, WC functions showed a relatively pronounced increase of 3.13%, while SC and WSF functions remained fundamentally stable.
2. The spatial patterns of the three KES exhibit distinct characteristics and remain relatively stable. High-value zones for WC are concentrated in the QTP, southern mountainous regions, and key forest zones of Northeast China. High-value areas for SC are primarily distributed across the Loess Plateau, QTP, Yunnan-Guizhou Plateau, and the hilly regions of Southeast China. Meanwhile, WSF services are highly concentrated in northern arid and semi-arid regions and the QTP. Collectively, these regions constitute the core barriers of the national ecological security pattern.
3. Results from the KES degradation early-warning model, developed based on relative change rates, indicate that nearly half of the counties nationwide (48.96%) are categorized as “no alert,” while over one-third (36.97%) are under “light alert.” Counties in “moderate alert” and “severe alert” states account for smaller proportions, at 12.26% and 1.83%, respectively. Severe-alert counties exhibit a highly concentrated distribution, primarily localized in the extreme arid zones of Northwest China and the karst mountainous and hilly regions of Southwest China. The alerts in these critical areas are predominantly driven by the substantial degradation of SC services.

This study innovatively develops a fact-based early-warning model for the degradation of KES. Characterized by a clear workflow, streamlined computations, and multi-scale applicability, this approach provides an effective tool for the rapid and intuitive identification of ecological degradation risk areas from an administrative perspective. Beyond this practical utility, the findings not only contribute to a deeper scientific understanding of the effectiveness of ecological protection in the initial phase of China’s ecological civilization construction but also offer direct scientific support for differentiated and precise decision-making in territorial spatial ecological conservation and restoration.

## Data Availability

The data that support the findings of this study are available from the Resource and Environment Science Data Centre of the Chinese Academy of Sciences but restrictions apply to the availability of these data, which were used under license for the current study, and so are not publicly available. Data are however available from the authors upon reasonable request and with permission of the Resource and Environment Science Data Centre of the Chinese Academy of Sciences.

## Abbreviations

ES: Ecosystem Services
WC: Water Conservation
SC: Soil Conservation
WSF: Windbreak and Sand Fixation
KES: Key Ecosystem Services
QTP: Qinghai-Tibet Plateau

## References

[1] Zhou, Y. et al. A bibliographic review of the relationship between ecosystem services and human well-being. *Environ. Dev. Sustain.* **27**, 25965–25992. 10.1007/s10668-024-04791-3 (2025).

[2] Costanza, R. et al. Changes in the global value of ecosystem services. *Global Environ. Change*. **26**, 152–158. 10.1016/j.gloenvcha.2014.04.002 (2014).

[3] Keith, D. A. et al. A function-based typology for Earth’s ecosystems. *Nature* **610**, 513–518. 10.1038/s41586-022-05318-4 (2022). 36224387 10.1038/s41586-022-05318-4 PMC9581774

[4] MA. *Ecosystems and Human Well-Being* (Island, 2005).

[5] Zhou, G. et al. Resistance of ecosystem services to global change weakened by increasing number of environmental stressors. *Nat. Geosci.* **17**, 882–888. 10.1038/s41561-024-01518-x (2024).

[6] Li, G. et al. Mixed effectiveness of global protected areas in resisting habitat loss. *Nat. Commun.* **15**, 8389. 10.1038/s41467-024-52693-9 (2024). 39333073 10.1038/s41467-024-52693-9 PMC11437083

[7] Wang, J. et al. The role of human activity in decreasing ecologically sound land use in China. *Land. Degrad. Dev.* **29**, 446–460. 10.1002/ldr.2874 (2018).

[8] Kong, W. et al. The neglected cost: Ecosystem services loss due to urban expansion in China from a triple-coupling perspective. *Environ. Impact Assess. Rev.* **112**, 107827. 10.1016/j.eiar.2025.107827 (2025).

[9] Bryan, B. A. et al. China’s response to a national land-system sustainability emergency. *Nature* **559**, 193–204. 10.1038/s41586-018-0280-2 (2018). 29995865 10.1038/s41586-018-0280-2

[10] Yu, C. et al. Assessment of the effectiveness of China’s protected areas in enhancing ecosystem services. *Ecosyst. Serv.* **65**, 101588. 10.1016/j.ecoser.2023.101588 (2024).

[11] Zhang, J. et al. Integrating SDGs into China’s ecological civilization construction: Practical indicators and progress assessment. *Ecol. Indic.* **169**, 112921. 10.1016/j.ecolind.2024.112921 (2024).

[12] Li, Z. et al. Changes in the ecosystem service importance of the seven major river basins in China during the implementation of the Millennium development goals (2000–2015) and sustainable development goals (2015–2020). *J. Clean. Prod.* **433**, 139787. 10.1016/j.jclepro.2023.139787 (2023).

[13] Xiong, C., Xu, H. & Tian, Y. Assessment of ecosystem service value in China from the perspective of spatial heterogeneity. *Ecol. Indic.* **159**, 111707. 10.1016/j.ecolind.2024.111707 (2024).

[14] Chen, G. et al. Changes in water conservation and possible causes in the Yellow River Basin of China during the recent four decades. *J. Hydrol.* **637**, 131314. 10.1016/j.jhydrol.2024.131314 (2024).

[15] Qiao, X. et al. Assessing current and future soil erosion under changing land use based on InVEST and FLUS models in the Yihe River Basin, North China. *Int. Soil. Water Conserv.* **12**, 298–312. 10.1016/j.iswcr.2023.07.001 (2024).

[16] Wang, Q. et al. Impact of ecological governance policies on county ecosystem change in national key ecological functional zones: A case study of Tianzhu County, Gansu Province. *Ecol. Indic.* **154**, 110748. 10.1016/j.ecolind.2023.110748 (2023).

[17] Yuan, M. et al. Revealing the causes of ecosystem stability changes in the Northern Sand Prevention Belt of China over the past twenty years. *Environ. Sustain. Ind.* **28**, 101017. 10.1016/j.indic.2025.101017 (2025).

[18] Xiao, Y. & Ouyang, Z. Spatial-temporal Patterns and Driving Forces of Water Retention Service in China. *Chin. Geogr. Sci.* **29**, 100–111. 10.1007/s11769-018-0984-0 (2019).

[19] Yang, X., Zhang, L., Hou, M., Wu, F. & Ju, Y. Tracing the evolution of soil conservation in China: A review of historical insights and modern practices. *Int. Soil. Water Conserv.*10.1016/j.iswcr.2025.10.008 (2025).

[20] Zhang, Z. & Huisingh, D. Combating desertification in China: Monitoring, control, management and revegetation. *J. Clean. Prod.* **182**, 765–775. 10.1016/j.jclepro.2018.01.233 (2018).

[21] National Development and Reform Commission. National and Regional Major Function Zone Planning (Part 1). 779. (2015).

[22] Bai, Y. et al. Developing China’s Ecological Redline Policy using ecosystem services assessments for land use planning. *Nat. Commun.* **9**, 3034. 10.1038/s41467-018-05306-1 (2018). 30072771 10.1038/s41467-018-05306-1 PMC6072749

[23] Costanza, R. et al. The value of the world’s ecosystem services and natural capital. *Nature* **387**, 253–260. 10.1038/387253a0 (1997).

[24] Wang, S., Liu, Y., Li, Y. & Bojie, F. Research progress on the ecosystem services on the Loess Plateau during the recent 20 years. *Acta Ecol. Sin.* **43**, 26–37. 10.5846/stxb202204291190 (2023). [Chinese].

[25] Ying, X., Wu, T., Su, B., Zhu, X. & Xiao, Y. Discussion on the evaluation method of cryosphere services. *J. Glaciology Geocryology*. **41**, 1271–1280. 10.7522/j.issn.1000-0240.2019.0537 (2019). [Chinese].

[26] Zhang, C., Su, B., Beckmann, M. & Volk, M. Emergy-based evaluation of ecosystem services: Progress and perspectives. *Renew. Sust Energ. Rev.* **192**, 114201. 10.1016/j.rser.2023.114201 (2024).

[27] Huang, L., Cao, W., Xu, X., Fan, J. & Wang, J. Linking the benefits of ecosystem services to sustainable spatial planning of ecological conservation strategies. *J. Environ. Manage.* **222**, 385–395. 10.1016/j.jenvman.2018.05.066 (2018). 29870967 10.1016/j.jenvman.2018.05.066

[28] Li, H. et al. A framework for dynamic assessment of soil erosion and detection of driving factors in alpine grassland ecosystems using the RUSLE-InVEST (SDR) model and Geodetector: A case study of the source region of the Yellow River. *Ecol. Inf.* **85**, 102928. 10.1016/j.ecoinf.2024.102928 (2025).

[29] Lin, X., Zhang, Y. & Ouyang, Z. Constraint effects of ecosystem health changes on wind erosion prevention service on the Tibetan Plateau. *J. Environ. Manage.* **395**, 127817. 10.1016/j.jenvman.2025.127817 (2025). 41166951 10.1016/j.jenvman.2025.127817

[30] Wang, Q. & Bai, X. Spatiotemporal characteristics of human activity and land use on ecosystem service functions in mountainous areas of Northeast Guizhou, Southwest China. *Ecol. Eng.* **212**, 107473. 10.1016/j.ecoleng.2024.107473 (2025).

[31] Wang, Y. et al. Spatiotemporal variations in water conservation function of the Tibetan Plateau under climate change based on InVEST model. *J. Hydrol. Reg. Stud.* **41**, 101064. 10.1016/j.ejrh.2022.101064 (2022).

[32] Li, X. et al. Exploring ecosystem service dynamics and drivers in the upper and middle Yellow River Basin under large-scale ecological restoration. *Ecol. Eng.* **217**, 107643. 10.1016/j.ecoleng.2025.107643 (2025).

[33] Meng, Y. et al. Ecosystem services in the Northern Tianshan Urban Agglomeration: Nonlinear responses to natural and human factors and threshold-based spatial optimization strategies. *J. Hydrol. Reg. Stud.* **61**, 102682. 10.1016/j.ejrh.2025.102682 (2025).

[34] Yao, S., Li, Y., Quan, X., Huang, G. & Xu, J. Exploring the trade-offs and synergies among ecosystem services to support ecological management in the Yangtze River Delta Urban Agglomeration. *J. Environ. Manage.* **393**, 127028. 10.1016/j.jenvman.2025.127028 (2025). 40858080 10.1016/j.jenvman.2025.127028

[35] Chen, X., Huang, L. & Zhang, C. Spatiotemporal evolution and trade-offs/synergies of ecosystem services in Hubei Province. *Sci. Rep.* **15**, 35697. 10.1038/s41598-025-19570-x (2025). 41083666 10.1038/s41598-025-19570-x PMC12518542

[36] Wang, C. et al. Driving factors of ecosystem services and their trade-offs and synergies in different land use functional zones: A case study of Shanxi Province, China. *Environ. Sustain. Ind.* **28**, 101011. 10.1016/j.indic.2025.101011 (2025).

[37] Chen, W., Chi, G. & Li, J. The spatial association of ecosystem services with land use and land cover change at the county level in China, 1995–2015. *Sci. Total Environ.* **669**, 459–470. 10.1016/j.scitotenv.2019.03.139 (2019). 30884268 10.1016/j.scitotenv.2019.03.139

[38] Wu, Q., Cao, Y., Su, D. & Cao, Y. A multi-scale framework for understanding spatial scale effects on ecosystem service heterogeneity, interactions, drivers and their socio-ecological impact pathways for adaptive management. *J. Clean. Prod.* **516**, 145757. 10.1016/j.jclepro.2025.145757 (2025).

[39] Che, J., Zhu, X. & Niu, X. Spatio-temporal patterns and driving mechanisms of ecosystem services in mountainous regions: A multi-scale analysis of the Yanshan-Taihang mountain area. *Sci. Rep.* **15**, 45521. 10.1038/s41598-025-29608-9 (2025). 41310023 10.1038/s41598-025-29608-9 PMC12749296

[40] Niu, Z. et al. Spatio-temporal characteristic of terrestrial ecosystem services and their tradeoffs and synergies in China from 2000 to 2018 based on a process model. *Acta Ecol. Sin.* **43**, 496–509. 10.5846/stxb202110112855 (2023). [Chinese].

[41] Xiong, M., Li, J. & Sun, R. Unraveling the paradox of soil erosion and conservation: Insights from China. *Sci. Total Environ.* **955**, 177134. 10.1016/j.scitotenv.2024.177134 (2024). 39461519 10.1016/j.scitotenv.2024.177134

[42] Zhou, A., Zhao, W., Han, Y., Zhang, S. & Pereira, P. Effects and benefits of wind erosion prevention in China’s dryland and surrounding countries. *Catena* **251**, 108812. 10.1016/j.catena.2025.108812 (2025).

[43] Ouyang, Z. et al. Improvements in ecosystem services from investments in natural capital. *Science* **352**, 1455–1459. 10.1126/science.aaf2295 (2016). 27313045 10.1126/science.aaf2295

[44] Lei, K. et al. The spatiotemporal variation characteristics and influencing factors of ecosystem services in national parks. *Acta Ecol. Sin.* **45**, 11442–11462. 10.20103/j.stxb.202507301984 (2025). [Chinese].

[45] Gao, M., Hu, Y., Niu, S., Bai, Y. & Wang, J. The Impact of China’s National Key Ecological Function Areas Policy on Ecosystem Service Enhancement. *Land. Degrad. Dev.*10.1002/ldr.70289 (2025).

[46] Dong, Y. et al. Does unified management enhance ecosystem services in transboundary nature reserves? A spatio-temporal assessment of the Changbai Mountain National Nature Reserve. *J. Clean. Prod.* **534**, 147066. 10.1016/j.jclepro.2025.147066 (2025).

[47] Kang, P., Chen, W., Hou, Y. & Li, Y. Linking ecosystem services and ecosystem health to ecological risk assessment: A case study of the Beijing-Tianjin-Hebei urban agglomeration. *Sci. Total Environ.* **636**, 1442–1454. 10.1016/j.scitotenv.2018.04.427 (2018). 29913604 10.1016/j.scitotenv.2018.04.427

[48] Zhang, Z. et al. Long-term assessment of ecological risk dynamics in Wuhan, China: Multi-perspective spatiotemporal variation analysis. *Impact Assess. Rev.* **105**, 107372. 10.1016/j.eiar.2023.107372 (2024).

[49] Peng, Y., Welden, N. & Renaud, F. G. A framework for integrating ecosystem services indicators into vulnerability and risk assessments of deltaic social-ecological systems. *J. Environ. Manage.* **326**, 116682. 10.1016/j.jenvman.2022.116682 (2023). 36375428 10.1016/j.jenvman.2022.116682

[50] Jin, W. et al. Diagnosis of ecological security and the spatial heterogeneity of its driving factors in the mining-impacted watershed, based on ecosystem health-risk-services framework. *Ecol. Indic.* **167**, 112683. 10.1016/j.ecolind.2024.112683 (2024).

[51] Hu, Y. et al. Multi-scenario spatial optimization for future development in arid and semi-arid regions based on early warning of ecological risk. *Ecol. Modell*. **510**, 111291. 10.1016/j.ecolmodel.2025.111291 (2025).

[52] Deng, G. et al. Projecting the response of ecological risk to land use/land cover change in ecologically fragile regions. *Sci. Total Environ.* **914**, 169908. 10.1016/j.scitotenv.2024.169908 (2024). 38190905 10.1016/j.scitotenv.2024.169908

[53] Li, J., Chen, X., De Maeyer, P., Van de Voorde, T. & Li, Y. Ecological security warning in Central Asia: Integrating ecosystem services protection under SSPs-RCPs scenarios. *Sci. Total Environ.* **912**, 168698. 10.1016/j.scitotenv.2023.168698 (2024). 38040380 10.1016/j.scitotenv.2023.168698

[54] Reddy, C. S. et al. Conservation priorities of forest ecosystems: Evaluation of deforestation and degradation hotspots using geospatial techniques. *Ecol. Eng.* **91**, 333–342. 10.1016/j.ecoleng.2016.03.007 (2016).

[55] Sims, N. et al. *I.J.S.i. Good practice guidance. SDG Indicator 15.3.1, Proportion of Land That Is Degraded Over Total Land Area. Version 2.0* (United Nations Convention to Combat Desertification, 2021).

[56] Teich, I., Gonzalez Roglich, M., Corso, M. L. & García, C. L. Combining Earth Observations, Cloud Computing, and Expert Knowledge to Inform National Level Degradation Assessments in Support of the 2030 Development Agenda. *Remote Sens.* **11**, 2918. 10.3390/rs11242918 (2019).

[57] Liu, W., Zheng, J., Wang, Z., Li, R. & Wu T.-h. A bibliometric review of ecological research on the Qinghai–Tibet Plateau, 1990–2019. *Ecol. Inf.* **64**, 101337. 10.1016/j.ecoinf.2021.101337 (2021).

[58] Xu, J., Xiao, Y., Xie, G., Wang, Y. & Lei, G. Assessment of the benefit diffusion of windbreak and sand fixation service in National Key Ecological Function areas in China. *Aeolian Res.* **52**, 100728. 10.1016/j.aeolia.2021.100728 (2021).

[59] Wang, L., Zheng, H., Chen, Y. & Huang, B. Ecological redline policy strengthens sustainable development goals through the strict protection of multiple ecosystem services. *Global Ecol. Conserv.* **56**, e03306. 10.1016/j.gecco.2024.e03306 (2024).

[60] Zhang, M., Yu, S., Ye, Q. & Zhao, Z. The impact of vegetation-dominated landscape configurations on the spatiotemporal dynamics and driving mechanisms of ecosystem services in the loess plateau. *Environ. Dev. Sustain.*10.1007/s10668-025-06790-4 (2025).

[61] Hafeez, A. et al. Chapter 12 - Soil and water conservation under changing climate. In: Challenges and Solutions of Climate Impact on Agriculture. Fahad, (eds Adnan, S., Munir, M., Lal, I., Nawaz, R. & Saud, S.) T., and. Academic, 307–328. (2025).

[62] Bai, Y., Han, H. & Jian, Y. Review of ecosystem services in karst rocky desertification controls: status, challenges, and future directions. *Trop. Ecol.* **66**, 404–421. 10.1007/s42965-025-00394-9 (2025).

